# Integrating tuberculosis control into maternal and newborn health services during pregnancy and postpartum is an urgent priority

**DOI:** 10.1371/journal.pgph.0006241

**Published:** 2026-04-08

**Authors:** Uduak Okomo, Esin Nkereuwem, Hannah Blencowe, Aduragbemi Banke-Thomas, Toyin Togun

**Affiliations:** 1 Medical Research Council Unit The Gambia at the LSHTM, Fajara, The Gambia; 2 Centre for Maternal, Adolescent, Reproductive and Child Health, London School of Hygiene and Tropical Medicine, United Kingdom; 3 Faculty of Infectious and Tropical Diseases, London School of Hygiene and Tropical Medicine, London, United Kingdom; 4 Tuberculosis Centre, London School of Hygiene and Tropical Medicine, United Kingdom; 5 Faculty of Epidemiology and Population Health, London School of Hygiene and Tropical Medicine, London, United Kingdom; PLOS: Public Library of Science, UNITED STATES OF AMERICA

## An overlooked burden

Tuberculosis during pregnancy and postpartum continues to be under-recognised worldwide, despite bridging two major global health priorities: tuberculosis control and maternal and newborn survival. Recent estimates suggest that approximately 239,000 women contract tuberculosis during pregnancy each year, with an additional 97,600 cases occurring postpartum, affirming that the postpartum phase should not be viewed as merely incidental but as a significant extension of the period of risk [1]. These figures demonstrate that pregnancy-related tuberculosis constitutes a significant portion of the overall tuberculosis burden among women of reproductive age, particularly in high tuberculosis-incidence regions [2].

Tuberculosis during pregnancy is associated with adverse maternal outcomes such as anaemia, pre-eclampsia and eclampsia, postpartum haemorrhage, and death, as well as adverse perinatal results like preterm birth, low birth weight, stillbirth, and early neonatal death [3,4]. Also, tuberculosis co-infection in women of reproductive age living with human immunodeficiency virus (HIV) substantially increases the risk of both adverse pregnancy outcomes and mortality, which further underlines the intersection of tuberculosis with broader maternal health inequities [1]. In many high-burden settings, women at the highest risk of tuberculosis are also those affected by HIV, poverty, undernutrition, overcrowded living conditions, and limited access to healthcare [5].

## Missed opportunities for diagnosis across antenatal and postnatal care

Routine antenatal care does not reliably include systematic tuberculosis screening, even among women at high risk. Where screening is undertaken, it is often symptom-based, inconsistently applied, and poorly linked to confirmatory testing and treatment. Antenatal care is one of the few points at which large numbers of women have repeated contact with the health system [6], and current World Health Organization guideline recommends at least eight antenatal contacts during pregnancy. These repeated visits create multiple opportunities to ask about symptoms, identify women at risk, investigate possible tuberculosis, and initiate treatment early. Yet in many settings, these contacts are not used effectively for case-finding, and as such are missed opportunities when timely detection and treatment could improve outcomes for both women.

Undiagnosed maternal tuberculosis at the time of delivery represents a missed opportunity to identify newborns exposed to tuberculosis and to initiate early clinical assessment, follow-up and preventive and/or therapeutic interventions as appropriate [4]. While congenital tuberculosis is rare, the greater risk to the newborn is postnatal exposure after birth from untreated maternal disease. During the first weeks of life, immature cell-mediated immunity places infants at high risk not only of acquiring infection but also of rapid progression to severe disseminated disease [7]. This contributes to preventable neonatal morbidity and mortality. In many high-burden settings, newborns receive Bacille Calmette-Guérin vaccination shortly after birth as part of routine immunisation policy [8]. However, neonatal vaccination does not remove the need for maternal case detection and appropriate management of mother-infant exposure.

Structured tuberculosis assessment is even less well established within postnatal care. In many healthcare systems, postnatal care is often one of the weakest links in maternal health services, even though significant morbidity and mortality occur during this phase [6]. Tuberculosis contributes to this overlooked burden; tuberculosis occurring after delivery may be missed because symptoms such as weight loss, fatigue, reduced appetite, and general weakness can be attributed to postpartum recovery or the demands of caring for a newborn.

## Where health systems are falling short

During pregnancy and in the postpartum period, data are not systematically recorded in many national tuberculosis surveillance platforms. As a result, programmes often cannot quantify the burden of tuberculosis among pregnant and postpartum women, assess patterns of diagnosis, monitor treatment outcomes, or evaluate the effectiveness of interventions in this population. The absence of pregnancy-disaggregated tuberculosis data limits both programmatic visibility and accountability. Although modelling studies may provide estimates of the burden in the absence of routine tuberculosis notification data, they should not substitute for surveillance systems that can directly and consistently identify pregnancy-associated tuberculosis.

There are also persistent gaps in evidence for clinical management. Pregnant and lactating women have historically been excluded from tuberculosis therapeutic and preventive trials, resulting in limited evidence on drug safety, pharmacokinetics, tolerability, and effectiveness during pregnancy and breastfeeding [9]. This exclusion has constrained the evidence base available to clinicians and policymakers and has slowed the development of context-specific guidance for women in the reproductive period. The recent World Health Organization consensus statement calling for the optimal and early inclusion of pregnant and lactating women in tuberculosis research reflects growing recognition that their systematic exclusion is neither scientifically justified nor ethically sustainable [10].

## What must happen next

**Fig 1:**
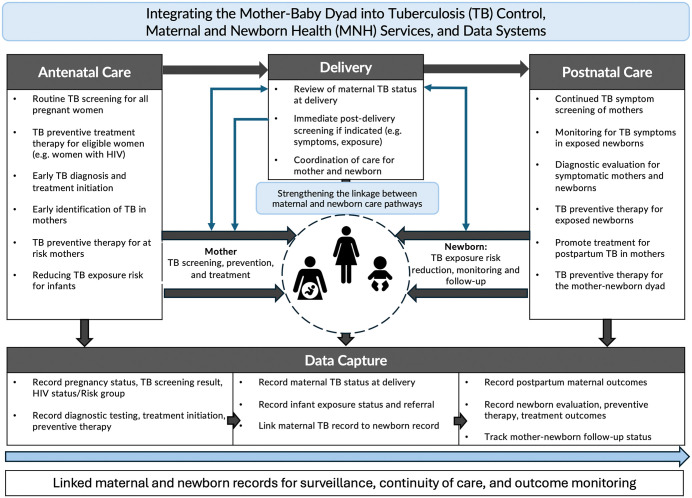
Conceptual framework showing opportunities to integrate the mother–infant dyad into tuberculosis care and routine data capture across antenatal, delivery, and postnatal care, with linkage of maternal and infant records to support continuity of care, surveillance, and outcome monitoring.

A stronger programmatic response is now needed. Tuberculosis screening, diagnostic evaluation, and preventive therapy should be more deliberately integrated into antenatal and postnatal care in high-burden settings, particularly for women living with HIV and other high-risk groups [11]. Such integration must extend beyond policy statements to include clear referral pathways, access to appropriate diagnostics, availability of treatment, and follow-up mechanisms. Routine data systems should capture pregnancy and postpartum status within tuberculosis notifications as a minimum reporting standard [**Fig 1**]. Greater attention should also be given to maintaining the mother-infant dyad in both clinical care and information systems, so that maternal diagnosis informs newborn risk assessment and follow-up.

These actions will require stronger alignment between tuberculosis programmes and maternal and newborn health services, supported by interdisciplinary collaboration that brings together maternal and newborn health specialists, tuberculosis experts, and implementation researchers to develop, test, and refine integration strategies and to address the barriers that continue to undermine implementation in practice, alongside sustained domestic investment. In many settings, integration is widely endorsed in principle but remains underfunded and weakly operationalised in practice. Yet in regions where maternal mortality remains high and tuberculosis burden is substantial, failure to address tuberculosis across pregnancy and the postpartum period remains an important missed opportunity to reduce preventable morbidity and mortality.

Tuberculosis should not remain peripheral to maternal and newborn health services, nor should pregnancy and the postpartum period remain peripheral to tuberculosis control. A more integrated, adequately resourced, and evidence-informed approach that guarantees data linkage is required if the response to the clarion call of the World TB Day 2026, “*Yes! We can end TB”* [12] is to reach women and their newborns living in high-burden settings.
